# High Density Lipoprotein Cholesterol Efflux Capacity and Atherosclerosis in Cardiovascular Disease: Pathophysiological Aspects and Pharmacological Perspectives

**DOI:** 10.3390/cells10030574

**Published:** 2021-03-05

**Authors:** Maria Pia Adorni, Nicoletta Ronda, Franco Bernini, Francesca Zimetti

**Affiliations:** 1 Unit of Neurosciences, Department of Medicine and Surgery, University of Parma, 43125 Parma, Italy; mariapia.adorni@unipr.it; 2 Department of Food and Drug, University of Parma, 43124 Parma, Italy; nicoletta.ronda@unipr.it (N.R.); francesca.zimetti@unipr.it (F.Z.)

**Keywords:** reverse cholesterol transport, high density lipoprotein, cholesterol efflux capacity, atherosclerosis, cardiovascular disease

## Abstract

Over the years, the relationship between high-density lipoprotein (HDL) and atherosclerosis, initially highlighted by the Framingham study, has been revealed to be extremely complex, due to the multiple HDL functions involved in atheroprotection. Among them, HDL cholesterol efflux capacity (CEC), the ability of HDL to promote cell cholesterol efflux from cells, has emerged as a better predictor of cardiovascular (CV) risk compared to merely plasma HDL-cholesterol (HDL-C) levels. HDL CEC is impaired in many genetic and pathological conditions associated to high CV risk such as dyslipidemia, chronic kidney disease, diabetes, inflammatory and autoimmune diseases, endocrine disorders, etc. The present review describes the current knowledge on HDL CEC modifications in these conditions, focusing on the most recent human studies and on genetic and pathophysiologic aspects. In addition, the most relevant strategies possibly modulating HDL CEC, including lifestyle modifications, as well as nutraceutical and pharmacological interventions, will be discussed. The objective of this review is to help understanding whether, from the current evidence, HDL CEC may be considered as a valid biomarker of CV risk and a potential pharmacological target for novel therapeutic approaches.

## 1. Introduction

High-density lipoprotein cholesterol (HDL-C) has been considered for years the “good” cholesterol, as suggested by the inverse correlation between its plasma levels and the cardiovascular (CV) risk, as shown by the epidemiology [1,2]. However, the relationship between HDL and atherosclerosis was found to be much more complex compared, for example, to that observed for low density lipoproteins (LDL). Firstly, a recent study has revealed a paradoxical association between very high HDL-C levels and increased CV disease (CVD) [3]. In addition, the failure of the trials testing the HDL-C raising drugs, together with the results of genetic studies on several polymorphisms or rare mutations, have led to some shadow being cast on the “HDL hypothesis” [4,5,6].

In parallel, a growing interest pointed to the importance of the HDL function in the context of atheroprotection. In facts, HDL possess several functions, among which the best known is the ability to promote the first step of the reverse cholesterol transport (RCT) process, namely the cholesterol efflux from the macrophages of the arterial wall [7,8].

Cell cholesterol efflux may occur through multiple mechanisms including aqueous diffusion, as well as facilitated and active transport both promoted by specific membrane proteins, such as the scavenger receptor class B, type I (SR-BI) and members of the ATP binding cassette (ABC) transporter family, ABCG1 and ABCA1, respectively [9]. The ability of HDL to promote cell cholesterol efflux (cholesterol efflux capacity, CEC) appears to be more related to HDL size and composition in terms of proteins and lipids, than to HDL-C plasma levels. CEC is usually evaluated by radioisotopic or fluorimetric bioassays in which cells are exposed to the HDL serum fraction, obtained by depletion of serum of the apolipoprotein (apo)B-lipoproteins. Such evaluation has emerged as a potentially accurate estimate of the RCT efficiency in humans [10].

The clinical relevance of HDL CEC is well highlighted by many studies, in which an inverse relationship was detected between CEC and the prevalence of atherosclerosis, as well as the incidence of CV events, occurring independently of plasma HDL-C levels [11]. In addition, CEC has been found to be impaired in several genetic disorders leading to dyslipidemia, as well as in specific pathological conditions associated to higher CV risk, such as chronic kidney disease, diabetes, inflammatory and autoimmune diseases, endocrine disorders etc.

Within this context, the present comprehensive review aims to critically dissect out the complex relationship between HDL CEC and atherosclerosis in conditions at high CV risk, focusing on the most recent studies conducted in human populations and taking into consideration genetic and pathophysiologic aspects. In addition, the most relevant strategies modulating HDL CEC will be described, including lifestyle modifications, as well as nutraceutical and pharmacological interventions. The final objective of this review is to help understanding whether HDL CEC may be effective as a biomarker of CVD and as a concrete pharmacological target for novel therapeutic approaches.

## 2. HDL and RCT

HDL was discovered in the 1950s and in 1960s the Framingham Heart Study reported the inverse relationship between plasma levels of these lipoproteins and atherosclerosis [1]. This milestone discovery encouraged investigations of the mechanism by which HDL could exert their antiatherogenic properties, leading to the identification of the RCT process [12]. RCT, proposed for the first time by Glomset, is the key physiological pathway by which excess cholesterol is removed from the peripheral tissues to the liver for the final excretion into the bile and feces [12]. RCT may prevent the formation and progression of atherosclerotic plaque through the HDL-mediated removal of cholesterol from the arterial wall, identified as the main anti-atherogenic mechanisms of HDL.

This process could be outlined in three phases:

1. Cellular cholesterol efflux from macrophages;
2. HDL remodelling in plasma;
3. Cholesterol hepatic uptake and excretion.

### 2.1. Cellular Cholesterol Efflux from Macrophages

In the first step, cholesterol is initially moved from arterial macrophages to extracellular HDL [13]. This is the rate-limiting step of the entire RCT process [13] and plays a pivotal role in the maintenance of intracellular cholesterol homeostasis, crucial for macrophage function and viability. The excess of free cholesterol (FC) is toxic for cells, and this may be a very important factor considering that most peripheral cells and tissues (except for those of steroidogenic organs) cannot catabolize cholesterol. For this reason, macrophages protect themselves against FC accumulation by either transforming it to cholesteryl esters (CE) for intracellular storage or by effluxing it to extracellular acceptors such as HDL [14]. Cholesterol efflux mainly depends on macrophage cholesterol content, on the expression of various macrophage cholesterol transporters that mediate efflux and also on the features of the HDL acting as extracellular acceptors, mainly in terms of lipid and protein composition, as well as size [15,16]. Four main mechanisms have been described to mediate the release of cellular cholesterol in the extracellular space and they are described in detailed in Section 2.4.

### 2.2. HDL Remodeling in Plasma

In the subsequent RCT steps, cholesterol-enriched HDL may undergo remodeling in size and composition through the activity of the two enzymes lecithin-cholesterol acyltransferase (LCAT) and cholesteryl ester transfer protein (CETP). LCAT, synthetized in the liver, is the only enzyme able of esterifying FC of HDL to CE, transforming nascent discoidal particles in spherical HDL. The preferential lipoprotein substrates for LCAT are the discoidal preβ-HDL, produced through the initial interaction of lipid-free apolipoprotein A-I (apoA-I) with the ABCA1 transporter, with consequent cellular efflux of FC and phospholipids [17]. By esterifying HDL cholesterol, the enzyme LCAT helps to maintain the unesterified cholesterol gradient between the cell membrane and the extracellular acceptors, determining a constant flux of cholesterol from periphery to circulating lipoproteins and avoiding cholesterol reuptake by cells [18]. Despite its central function in HDL remodeling and maturation, the role of LCAT in the pathogenesis of atherosclerosis is still debated. In mice, the overexpression of human LCAT increased circulating HDL levels, but did not enhance macrophage RCT. Moreover, a significant macrophage RCT emerged in LCAT-deficient mice despite extremely low plasma levels of HDL-C [19]. Existing data in humans are controversial, but markedly support the idea that decreased LCAT concentration and activity, despite reducing HDL levels, are not related with the pathogenesis of atherosclerosis [17]. In particular, a study by our group demonstrated that serum from LCAT deficiency patients display increased ABCA1-mediated capacity to promote cholesterol efflux compared to control subjects, due to the presence of high levels of preβ-HDL [20].

The second enzyme playing a key role in RCT is CETP. This enzyme is an hydrophobic glycoprotein mainly produced by the liver, circulating in plasma largely bound to HDL [21]. It promotes the net mass transfer of CE from antiatherogenic HDL to proatherogenic apolipoprotein B (apoB)-containing lipoproteins, in exchange with triglycerides (TG). Human studies have generally supported the idea that CETP deficiency, associated with increased HDL and apoA-I levels, is antiatherogenic. This hypothesis has paved the way for developing CETP inhibitors as a possible strategy to raise HDL levels in humans, to reduce atherosclerosis and to treat CVD [22]. In human studies, CETP inhibition indeed induced an increase in HDL-C together with a decrease in the non-HDL-C. Nevertheless, clinical studies have failed in proving their capacity to reduce CV risk [23].

### 2.3. Cholesterol Hepatic Uptake and Excretion

In the third step of RCT, free and esterified HDL-derived cholesterol are uptaken by hepatocytes through the transporter SR-BI, generating cholesterol-poor HDL that can be recycled [24]. Moreover, in addition to the major pathway via SR-BI, under some conditions, HDL can be recognized also by LDL receptors (LDLr) in the liver, as observed in mice [25]. In addition, a recent study supported a novel concept suggesting that, at least in mice, a shift in macrophage-derived unesterified cholesterol from HDL to LDL provides a significant route for macrophage cholesterol to reach the liver via the hepatic LDLr [26].

After hepatic uptake, cholesterol can be eliminated into the bile as neutral sterols via the transporters ABCG5/ABCG8 or through ABCA1, after conversion into bile acids, and excreted via faeces [27]. For many years, the hepatobiliary route has been considered the only way for cholesterol excretion. Conversely, in recent years another metabolic pathway has been discovered. This mechanism, known as trans-intestinal cholesterol excretion, takes place directly from plasma to the lumen of the intestine and markedly contributes to the total fecal neutral sterol excretion [28].

### 2.4. Mechanisms of Cell Cholesterol Efflux

**Figure 1:**
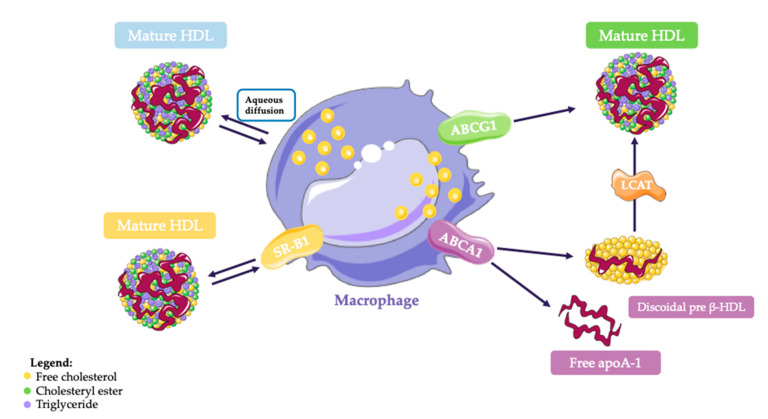
Mechanisms of cellular cholesterol efflux. This scheme shows the different pathways involved in macrophage cholesterol efflux and the specific high-density lipoprotein (HDL) subclasses acting as cholesterol acceptors. Cholesterol efflux occurs by four independent routes, including aqueous diffusion (AD), scavenger receptor B1 (SR-BI), ATP-binding membrane cassette transporter A1 (ABCA1) and G1 (ABCG1). The AD and SR-BI pathways consist of bidirectional flux of cholesterol between mature HDL particles and the cell plasma membrane and, as such, cholesterol transfer is driven by the cholesterol concentration gradient. Furthermore, cholesterol efflux can be unidirectionally and actively transported through the transporter ABCA1 to lipid free apoA-1 or discoidal pre β-HDL or through the transporter ABCG1 to mature HDL particles.

As mentioned before, the first step of the RCT process consists of cellular cholesterol efflux from macrophages of the arterial wall to HDL [7,14]. The efflux process consists of four main pathways (Figure 1):

1. The aqueous diffusion (AD)-mediated efflux. It is a pathway that depends on the cholesterol gradient between cells and acceptors [9];
2. The Scavenger type B class 1 (SR-BI)-mediated efflux. It facilitates the diffusion of FC to mature HDL according to a gradient and it is bidirectional (Figure 1) [29].
3. The ABCA1-mediated efflux.
4. The ABCG1-mediated efflux.

The cholesterol efflux mediated by these two transporters is unidirectional towards serum acceptors such as lipid free apoA-I and mature HDL, respectively (Figure 1) [30,31]. In addition, it has been reported that small discoidal preβ-HDL can act as cholesterol acceptors for both the mechanisms [32].

Since all the above described efflux pathways are active in macrophages, a study assessed the actual contributions of each of them. Adorni et al. conducted a study on cholesterol-loaded mouse peritoneal macrophages, showing that the removal of ABCA1 reduced efflux by 50%, while approximately 20% of the cholesterol efflux was attributable to ABCG1. Moreover, it was demonstrated that AD is one of the major contributors to efflux in macrophages while the SR-BI contribution to efflux is small [33], even though this may be dependent on the initial macrophage cholesterol content, as well as on the absence or presence of the other transporters [34,35].

In the AD process, FC crosses the cell membrane towards extracellular mature HDL. The speed of the process depends on acceptor size affecting the collision with the cholesterol molecules present in the aqueous phase. The AD occurs in all cells and is a relatively inefficient mechanism compared to the active processes [30].

#### 2.4.1. SR-BI

The SR-BI receptor is a member of the CD36 family of proteins with a large extracellular N-glycosylated domain (408 aa) containing a cysteine-rich region. A transmembrane domain with short extensions into the cytoplasm binds the extracellular domain at the N- and C-terminal tails. SR-BI is well conserved between species and is expressed in many cell types, including intestine, macrophages, endothelial cells, smooth muscle cells, keratinocytes, adipocytes, and placenta [29]. The expression in macrophages is under control of the peroxisome proliferator-activated receptors alpha and gamma (PPAR-α and -γ) [30]. SR-BI promotes a FC bidirectional flux between cells and mature HDL particles, occurring through a cholesterol concentration gradient [29]. Several studies on mouse models have shown that SR-BI overexpression in the liver decreases atherosclerosis, whereas partial or total loss of SR-BI increases atherosclerosis [29]. Consistently, subjects carrying the P376L variant for the gene encoding for SR-BI have abnormally increased plasma levels of HDL-C paralleled to an increased risk of coronary heart disease (CAD) [36], further supporting the assumption that HDL-C level does not linearly correlate with the CVD risk [37]. Beyond the role in cholesterol efflux, SR-BI exerts its atheroprotective activity by regulating macrophage inflammation through activation of Akt and the suppression of NF-κB after the interaction with HDL [29].

#### 2.4.2. ABCA1

ABCA1 belongs to the ATP-binding cassette transporters family, which consists of 49 members divided into seven subfamilies, from A to G, all using ATP as a source of energy [38]. ABCA1 is an integral membrane protein composed of 2261 amino-acids, containing 2 transmembrane domains, each of them composed of six transmembrane filaments with one nucleotide-binding domain and one small regulatory domain. This transporter was discovered in 1999 in patients affected by Tangier disease, a rare condition that leads to a dramatic decrease in HDL-C plasma concentration, accumulation of foam cells in various tissues, peripheral neuropathy and a moderate increase in atherosclerosis. It was found that several mutations in the ABCA1 gene are responsible for this disease in humans [39]. ABCA1 controls the transfer of cholesterol and phospholipids to apoA-I, a process which leads to the formation of preβ-migrating HDL particles [40]. In a previous study, it has been suggested that ABCA1 interacts with the monomolecular, lipid-free/lipid-poor apoA-I and with a discoidal HDL particle with a diameter of 7.8 nm, containing two apoA-I molecules [32]. In addition to apoA-I, other apolipoproteins such as apolipoprotein E (apoE), apoA-II or apoA-IV can serve as ABCA1 acceptors [41,42]. The efflux mediated by ABCA1 is unidirectional and its limiting step is the binding of apoA-I to the cellular membrane with a direct interaction with the transporter, followed by apoA-I-mediated solubilization of the plasma membrane domains, ending in the formation of discoidal nascent HDL particles [43]. A defective ABCA1 activity leaves apoA-I poorly lipidated, accelerating its elimination from the kidneys and eventually preventing the formation of HDL [44]. Epidemiological studies have suggested that genetic variations in ABCA1 are associated with CV risk independently of plasma levels of HDL cholesterol, as shown by Frikke-Schmidt and collaborators, who reported data on the association between common single nucleotide polymorphisms (SNP) on ABCA1 and risk of CVD mortality [45].

The regulation of ABCA1 gene transcription is promoted by adenosine cyclic monophosphate (cAMP), and phosphodiesterase inhibitors 4, which stimulate the gene transcription [46]. The post-transcriptional regulation of ABCA1 is promoted by the bond with apoA-I or apoE, which stabilizes the transporter preventing calpain-mediated degradation [43]. Macrophage expression of ABCA1 is transcriptionally regulated by nuclear receptors such as the Liver X Receptor (LXR) acting as heterodimer with the Retinoid X Receptor (RXR). Endogenous ligands for LXR are oxysterols generated by the oxidation of cholesterol. In particular, oxysterols and 9-cis-retinoic acid (9cRA) activate transcription by binding to LXR and RXR, respectively, to form heterodimers that bind to conserved LXR-responsive elements [43].

#### 2.4.3. ABCG1

ATP binding cassette transporter G1 (ABCG1) is a membrane half-transporter with a single nucleotide binding domain in the transmembrane domain, composed of six transmembrane helices, that is responsible for ATP binding and hydrolysis. The ABCG1 protein is expressed in many cell types as macrophages, neurons, astrocytes, endothelial, and epithelial cells, and in many tissues, such as the liver, intestine, kidney, spleen, lung, and brain [47]. ABCG1 forms homodimers or heterodimers in association with other ABC half-transporters (e.g., ABCG5/ABCG8) [47]. Differently from ABCA1, ABCG1 promotes efflux mainly to mature HDL [39]. In this regard, it seems that both ABCA1 and ABCG1 transporters can act in sequence: ABCA1 promotes the efflux of cholesterol and phospholipids to apoA-I or pre-β-migrating HDL, leading to the formation of lipidated discoidal HDL which, in turn, favor ABCG1-mediated efflux [39]. A previous study demonstrated that the ABCG1-mediated cell cholesterol efflux can be also driven by pre-β HDL particles with sizes ≥ 7.8 nm [32]. In addition, the activity of ABCG1 is partly nonspecific, as it can promote efflux not only to HDL but also to LDLs and to phospholipid vesicles [43]. It has been demonstrated that, differently to ABCA1, ABCG1-mediated efflux does not need lipoprotein binding to the cell surface. Two different mechanisms for ABCG1-mediated cholesterol efflux were proposed. The first mechanism, proposed by Small in 2003, suggests that ABCG1 facilitates protrusion of cholesterol from the membrane pool into the hydrophilic water layer lining the plasma membrane. Subsequently, cholesterol uptake by an acceptor, as HDL particles, occurs after transient collision [48]. A second model suggests that ABCG1 promotes changes in the organization of plasma membrane phospholipids that lead to a redistribution of sterols. This could result in an increased efflux of cholesterol out of the cell by AD because of plasma membrane enrichment in cholesterol [47]. ABCG1 expression is transcriptionally regulated through the LXR/RXR pathway, similarly to ABCA1 [43], although some authors suggested that LXR-induced cholesterol efflux to HDL is independent of ABCG1 expression [49]. ABCG1 seems to play a role in several metabolic disorders including diabetes, insulin resistance, obesity and CVD, although the results of mice and human studies are still controversial [50].

### 2.5. HDL CEC as an Index of RCT Efficiency

It is now well established from large-scale epidemiologic studies that HDL represent the main atheroprotective plasma lipid component; indeed, a 1 mg/dl increase in plasma HDL-C is associated with a 3–4% reduction in CV mortality [51]. However, pharmacological interventions aimed at raising HDL-C levels have not generally supported a beneficial effect on CV outcomes. Trials involving treatment with niacin or dalcetrapib failed to demonstrate clinical benefit despite increases in HDL-C [4,52]. Consistently, genetic studies showed that neither rare nor common genetic variants associated with HDL-C levels are strongly linked to CVD [53].

These findings together have reinforced the concept that HDL-C levels might be neither a proper predictor of CVD risk nor an appropriate therapeutic target. Thus, there has been great interest in determining whether another HDL metric may better capture its potential antiatherogenic effects. In this regard, HDL particles possess several potentially antiatherogenic properties including the antioxidative, the anti-inflammatory, and the antithrombotic [54]. However, the best-known activity of HDL is its central role in RCT. In this regard, the efficacy of HDL function in a single individual may be indeed estimated by measuring cholesterol efflux capacity (CEC) of HDL [55]. Several lines of evidence suggest that CEC correlates with HDL quality rather than quantity: for instance sera with similar HDL-C or apoA-I differ in their ability to promote macrophage efflux because of differences in the concentration of preβ-HDL [56], or subjects with the apoA-I_Milano_ mutation or with LCAT deficiency have high levels of circulating particles responsible for an efficient serum CEC despite very low levels of circulating HDL-C [20,57]. In support of the disconnection between CEC and HDL-C levels, an independent inverse association between HDL CEC and incident CV events has been shown independently of HDL-C circulating levels [58,59].

Thus, considering the growing evidence highlighting the better predictive power of HDL CEC rather than HDL-C levels, considerable efforts have been made to develop measurements of this parameter in different conditions related to CVD risk [8]. In this regard, the estimation of HDL CEC can be performed through different techniques [8], distinguishing the various mechanisms involved in cholesterol transport [33]. An overview with respect to the methods available for the measurement of CEC is well described in a recent review by Toh [55].

## 3. HDL CEC, Atherosclerosis and Cardiovascular Risk

A growing number of clinical studies have explored the relationship between CEC and CV risk, in the research of novel HDL-related biomarkers able to improve the CV risk stratification, as well as of novel pharmacological targets. Recently, the results of 20 studied have been summarized in a meta-analysis of cross-sectional and longitudinal studies. It was found that higher HDL CEC was inversely associated with the risk of CVD with a relative risk of 0.87 [11].

In this section, we will describe in detail and critically discuss the most relevant findings on the association between CEC and CV outcomes by distinguishing those obtained from cross-sectional and longitudinal settings.

### 3.1. Cross-Sectional Studies

The relevance of the HDL cholesterol efflux promoting function in atheroprotection was highlighted for the first time by Khera and colleagues in 2011 [37]. They evaluated HDL CEC in about 1000 subjects, demonstrating an inverse relationship between this functional parameter and subclinical atherosclerosis, as well as obstructive coronary artery disease, independent of plasma levels of HDL-C. Interestingly, they used a cell line of murine macrophages (J774) incubated with cAMP, in order to evaluate the so called total efflux, as result of the contribution of pathways of known relevance, but in which AD and ABCA1-mediated efflux play the major role [56].

Since then, a number of studies have been performed to deeply investigate the relationship between CEC and atherosclerosis [60,61,62], and the main results are shown in Table 1. These data have also been included in a meta-analysis reporting an inverse correlation between CEC and the prevalence of CVD events [63].

More recently, a case-control study analyzed 150 patients with acute coronary syndrome (ACS) and 110 healthy adults not presenting clinical CVD and not undergoing any lipid-lowering therapy. In ACS patients, HDL CEC was found to be reduced compared to controls and associated with higher odds of ACS presence, even after adjustment for confounding factors including plasma HDL-C levels [64]. Interestingly, after 6 months of standard therapy, HDL CEC was restored in ACS patients, further pointing to this functional parameter as a good candidate for CVD risk prediction.

The inverse correlation of CEC with atherosclerosis was assessed not only by measuring the total efflux from macrophages but also evaluating the single pathways relevant for cholesterol efflux, as described in Section 2.4. In detail, our research group detected an inverse association between the CEC specifically mediated by the transporter ABCA1 and the pulse wave velocity, an indicator of arterial stiffness [65], and a direct link between the flow-mediated dilation (FMD), index of endothelial function, and the CEC specifically promoted by the transporter ABCG1 [66].

In all the mentioned works emerged very clearly the independence between function and levels of HDL-C. Further strengthening this concept is the observation that, in patients with CVD, a reduced CEC was found compared to healthy controls, despite the former having higher plasma levels of HDL-C [67]. In addition, the cholesterol efflux promoting function of HDL may also contribute to explain the (U-shaped) relationship recently detected between HDL-C level and CVD risk [68]. It has been hypothesized that at the lowest HDL-C levels range, fewer HDL particles are able to mediate cell-cholesterol efflux, possibly explaining the high CV risk. On the other hand, the increased risk observed at very high HDL-C levels, may either be related to reduced HDL CEC, because of lower phospholipids (PL) [67], or CEC not further improved [66].

However, not all studies univocally agree on the predictive power of HDL CEC. For example, in 574 subjects with elevated risk of diabetes and CVD, HDL CEC were not associated with indexes of sub-clinical atherosclerosis, evaluated as intima-media thickness (IMT) and endothelial function, as well as the CV events prevalence [69]. Similarly, no association of HDL CEC with prevalent CV risk was detected in a cohort with stable angio-graphically confirmed CVD, after adjustment for traditional risk factors [70]. The reasons for these apparent discrepancies are currently unclear and they may be related to the individual characteristic of the analyzed subjects.

### 3.2. Longitudinal Studies

In several clinical trials, HDL CEC predicted future CV events [58,59,72,73,74,75,76,77,78] so it has been defined as a good marker of CVD risk in a recent roundtable discussion [10] (see Table 2 for the main results of longitudinal studies). The predictive power of HDL CEC on ACS incidence has also been recently evaluated in a prospective setting on a subset of high CV risk participants of the PREDIMED study. In these patients, higher levels of CEC were associated to lower odds of ACS, and specifically lower risk of MI (myocardial infarction), irrespective of other risk factors, including HDL-C [79]. The particularly significant association between CEC and MI risk clearly suggests that impaired CEC may predict a clinical setting characterized by lipid-rich vulnerable plaque, leading to acute events.

Concerning CV events, Ebtehaj S. and colleagues [71], by analyzing CEC in 705 cases and control subjects combined, observed an inverse association between CEC evaluated at the baseline and incident CV events, with an odds ratio of 0.73; (95% CI, 0.62–0.86; *p* < 0.001). Such association persisted also after adjustments for HDL-C and other potential confounding factors and is in agreement with two previous studies [58,59]. It is worth mentioning that, in the study by Ebtehaj S. et al., HDL CEC was reduced across subjects with initially lower or higher HDL-C concentrations. In addition, the main difference between this last and other studies is the cellular model that has been used to evaluate CEC (human THP-1 vs. murine J774 macrophages). Overall, these results indicate that CEC robustly was associated with future CVD, despite different protocols in terms of cell models.

Dysfunctional HDL with respect to CEC may also contribute to explain the association between other markers and CVD. For instance, by analyzing 2643 participants of the population-based cohort of the Dallas Heart Study, it was observed that impaired CEC can explain the association between GlycA, a predictive cardiometabolic biomarker reflecting the concentrations of five glycosylated acute phase proteins, and the incidence of CV events [80]. These data further strength the well documented interplay between HDL dysfunction, inflammation and CVD (see Section 4.3) [81].

As specified above for cross-sectional studies, the results on the predictive power of HDL CEC in prospective settings are also not univocal. For example, HDL CEC did not associate with CV or all-cause mortality. But predicted graft failure in a cohort of renal transplant recipients [82]. In addition, a study showed that a higher HDL CEC was associated with increased risk of incident CV events, also after adjustment for CV risk factors, including HDL-C and LDL-C [83]. Similarly, CEC was not associated with incident peripheral artery disease [84]. Even in this case, the reasons for these controversies are not clear and are worth further investigations. Moreover, in a posthoc analysis on the Justification for the Use of Statins in Prevention: an Intervention Trial Evaluating Rosuvastatin (JUPITER) trial, HDL CEC at baseline was not associated with incident CV events [72]. The authors justified the discrepancy with other studies with the baseline characteristics of the patients, namely the high level of C-reactive protein (CRP), index of a significant inflammatory status. However, an inverse relationship was instead observed in statin treated patients, possibly highlighting the relevance of HDL functionality in patients achieving very low levels of LDL-C.

In summary, although more research is still needed, it is possible to state that the majority of the studies highlight a robust and inverse link between HDL CEC and the CVD risk, suggesting CEC as a more representative biomarker, compared to merely plasma HDL-C concentrations, and as an attractive pharmacological target for innovative strategies to reduce CV risk. The relative impact of HDL functionality in CVD with respect to other metabolic factors remains to be established.

## 4. HDL CEC in Specific Conditions

### 4.1. HDL CEC in Genetic Disorders

The study of families with forms of extreme dyslipidaemia has led to the identification of genes involved in modulation of lipid and lipoprotein metabolism [85] and has clearly shown an association between dyslipidemia and clinical and subclinical atherosclerosis.

#### 4.1.1. Genic Alterations Leading to Hypoalphalipoproteinemia

ApoA-I (OMIM: 107680) is a 243-amino acid apolipoprotein encoded by *ApoA-I* gene, located on the 11q23-q24 chromosome; to date, more than 60 mutations have been described, with the majority associated with hypoalphalipoproteinemia [86].

Individuals carrying a genetic variant of apoA-I, apoA-I_Milano_ (A-I_M_), show a single aminoacidic substitution in position 173 of cysteine for arginine. apoA-I_Milano_ is the first identified and well characterized apoA-I mutation, leading to the formation of homodimers (A-I_M_/A-I_M_) and heterodimers with apoA-II [87]. All identified individuals are heterozygous for A-I_M_, presented elevated TG, a partial LCAT deficiency and low circulating LDL-C and HDL-C, in absence of an increased CV risk [88]. As discussed above, this apparent paradox is explained by the greater ability of serum from patients with A-I_M_ mutation to promote ABCA1-mediated CEC compared to serum from controls, due to the accumulation of a unique small A-I_M_/A-I_M_-containing HDL, with the same or even improved efficiency compared to normal apoA-I-containing preβ-HDL as a cell cholesterol acceptor via the ABCA1 pathway [57]. In parallel, the ABCG1- and SR-BI-mediated cholesterol efflux is lower compared to control subjects, as circulating levels of large HDL particles are reduced [57].

In a recent study, Nilsson et al. demonstrated that two amyloidogenic apoA-I variants, L75P or L174S, are more efficient as cholesterol acceptors from macrophages, despite an overall reduction in HDL-C levels, probably due to an increase in structural apoA-I flexibility compared to their native counterpart [89].

The V19L apoA-I mutation, recently identified in Icelanders, is associated with increased HDL-C and decreased CAD risk; rHDL-associated apoA-I(V19L) showed normal capacity to promote ABCA1- and ABCG1-mediated cholesterol efflux, but an increased (45%) ability to promote SR-BI-mediated cholesterol efflux, as a result of the increased ability of apoA-I(V19L) to bind to phospholipids, together with an enhanced thermodynamic stability [90].

On the other side, many apoA-I mutations are known to negatively affect CEC: a rare frameshift mutation (c.546_547delGC) forming a truncated protein (*p*.[L159Afs*20]) with an abnormal C-terminal domain, known as apoA-I_Guastalla_, leads to a great reduction in CEC, mainly through the ABCA1 transporter, probably reflecting the alterations in preβ-HDL subpopulation [91]. Similarly, subjects carrying apoA-I_Nichinan_, an apoA-I variant with a deletion of residue E253 in the C-terminal helical region, presented HDL with a reduced ABCA1-mediated cholesterol efflux capacity, as a result of the impaired apoA-I_Nichinan_-ABCA1 direct interaction and lipid affinity [92]. An heterozygous apoA-I missense mutation, known as apoA-I_Boston_ (c.517C.A, p.Arg149Ser), causing a reduction in HDL-C, free apoA-I levels and apoA-I content in very large HDL, leads to a reduction in total, ABCA1- and SR-BI-mediated CEC, probably as a consequence of the reduction in HDL mass [93]. Furthermore, three heterozygous missense mutations, Leu141Arg (apoA-I_Pisa_) [94], Arg160Leu (apoA-I_Oslo_) [95] and Pro165Arg [96], respectively, are associated with decreased LCAT activity, low HDL-C and decreased cholesterol efflux.

The *LCAT* gene (OMIM: 606967) is located on the 16q22 chromosome, encoding a 416-amino acid glycoprotein; mutations in this region are related to a partial or complete loss of enzymatic activity and hypoalphalipoproteinemia [97]. Subjects with homozygous LCAT mutation are characterized by extremely low HDL-C levels, alteration in HDL structure and distribution, with a selective reduction in large apoA-II-containing HDL and a predominance of preβ-HDL, while subjects with heterozygous LCAT mutation display a milder phenotype, with a reduction in HDL-C levels and increased β-HDL [98]. Bérard and colleagues analyzed total cholesterol efflux in a 34-year-old patient with LCAT deficiency caused by two mutations in exon 3 (Arg99→Cys and Tyr83 converted into a Stop codon), which was found to be as efficient as control plasma [99].

A deeper study of the relation between LCAT deficiency and CEC was carried out by our research group in a cohort of 41 homozygous and heterozygous carriers of LCAT mutations and specifically considering the different efflux pathways. We observed an increased ABCA1-mediated CEC in subjects with LCAT deficiency compared to controls, correlating with the increased content of preβ-HDL, with a gene-dose dependent effect. Consistently with the reduction in circulating HDL, both ABCG1-and SR-BI-mediated CEC were significantly reduced compared to controls, suggesting a compensatory increased ABCA1-mediated efflux for the reduced efflux through ABCG1 and SR-BI [20].

#### 4.1.2. Genic Alterations leading to Hyperalphalipoproteinaemia

The *CETP* gene (OMIM: 151670) is located on the 16q21 chromosome and encodes for a 476-amino acid hydrophobic protein. Genetic CETP deficiency is an extremely rare condition, causing familial hyperalphalipoproteinemia (HAL), and to date about 39 mutations have been found, especially in the Japanese population [100]. In general, the complete absence of CETP activity results in the total failure to transfer cholesteryl esters (CE) from HDL to other lipoproteins, resulting in the accumulation of CE in HDL, with a huge increase in their size; the heterozygous carriers of CETP deficiency, on the other side, show a milder phenotype, with a modest increase in apoA-I, HDL-C and HDL size [101]. Specifically, we previously analyzed CEC in a 63-year-old male with undetectable CETP activity, high HDL-C and apoA-I. CEC via ABCA1 was significantly reduced compared to serum from controls, while ABCG1- and SR-BI-mediated CEC was normal or even enhanced [102]. Miwa and colleagues recruited homozygous subjects completely lacking CETP activity and heterozygous subjects with defective CETP activity. HDL from homozygous subjects displayed a 38% increased ability to promote SR-BI-mediated cholesterol efflux compared to controls and, consistently, the isolated HDL_2_ showed a significantly 22.5% higher efflux capacity. On the other side, the CEC of serum with complete or partial CETP deficiency was preserved through the ABCA1 pathway [103]. Similarly, Plengpanich and colleagues analyzed CEC in a compound heterozygote patient showing that isolated HDL_2_ had a higher capacity to promote cholesterol efflux through SR-BI compared to those of control subjects [104]. The observations demonstrate that CETP deficiency is not correlated with atherosclerosis susceptibility in the absence of other major CV risk factors.

SR-BI (OMIM: 601040) is a 509-amino acid protein encoded by *SCARB1* gene on the 12q24.31 chromosome. Vergeer and colleagues identified a missense mutation (P297S) in subjects with increased HDL-C and a reduced serum CEC from macrophages, despite similar carotid artery IMT between carriers and noncarriers [105]. Hence, two other SR-BI mutations, S112F and T175A, localized in the same extracellular loop as P297S, are thought to be similarly less efficient in mediating cholesterol efflux, even if deep analyses are still needed [106].

Finally, our research group analyzed 20 subjects with HAL, related to mutations of the CETP, as well as of SR-BI and the LIPG genes. We found that, despite very high HDL-C levels, the HDL CEC through the transporters ABCA1 and ABCG1 was not further improved in these subjects compared to individuals with normal HDL-C levels [66], consistently with the concept that CEC is mainly a metric of HDL functionality rather than mirroring HDL plasma concentration. Similarly, in HAL patients also, the vascular function was not further improved compared to controls, suggesting no incremental atheroprotection at very high HDL-C levels.

#### 4.1.3. Genic Alterations leading to Hypercholesterolemia

Familial hypercholesterolemia (FH) is an autosomal dominant disorder representing the most common form of dyslipidaemia, with life-long elevated LDL-C, tendon xanthomas and extremely high risk of premature atherosclerotic CVD. Despite no direct mutation being involved in HDL-related genes, many studies demonstrated that FH is usually associated with quantitative and qualitative alterations in HDL properties, negatively affecting their antiatherogenic role in the RCT pathway [107]. Ottestad and colleagues reported indeed that FH patients with high TG-rich HDL_3_ display a reduction in HDL anti-inflammatory properties and ability to promote cholesterol efflux from macrophages [108]. Similarly, Bellanger and colleagues showed that large HDL_2_ particles isolated from 12 FH patients receiving an aggressive cholesterol-lowering therapy, but still with a marked type IIa hypercholesterolemic phenotype, also display a reduced CEC both through ABCG1- and SR-BI-mediated pathways, inversely correlating with carotid IMT [109]. Again, Versmissen and colleagues compared male FH subjects with or CHD (*n* = 7 and 6, respectively) and their non-FH sibling: plasma from FH patients without CHD displayed a higher CEC compared to plasma from FH patients that experienced CHD, with respect to their non-FH sibling, and alteration in HDL composition, such as the levels of cholesterol, S1P and apoM [110]. A wider study considered 227 heterozygous FH patients, mostly carrying LDLR or PCSK9 mutations, with or without atherosclerotic CVD, showing a decreased CEC in patients with CVD risk, despite the cholesterol lowering therapy. An inverse relationship was also observed between CEC and carotid IMT, as well as Achilles tendon thickness; moreover, patients with corneal arcus displayed a reduced CEC, after adjustment for age, sex and CV risk factors [61].

Lysosomal acid lipase (LAL; OMIM: 613497) is the enzyme responsible for acidic hydrolysis of cholesteryl esters and TG from lipoproteins to lysosomes, encoded by *LIPA* gene on the 10q23.31 chromosome [111]. Mutations in *LIPA* cause LAL deficiency, a very rare disorder with two recognized phenotypes: Wolman disease, characterized by the complete absence of LAL activity and high and premature mortality rate, and Cholesteryl Ester Storage Disease (CESD), with a residual 5–10% LAL activity accompanied by high transaminases, hepatomegaly, liver fibrosis, dyslipidemia (high LDL-C and low HDL-C), as well as premature atherosclerosis and CVD [112]. Our research group highlighted serum CEC impairment in three CESD patients compared to control subjects, considering the specific membrane cholesterol transporters ABCA1, ABCG1 and SR-BI, and reflecting the reduction in HDL-C levels as well as the observed HDL remodeling [113].

#### 4.1.4. Genic Alterations leading to Hypotriglyceridemia

Angiopoietin-like 3 protein (ANGPTL3; OMIM: 604774) is encoded by *ANGPTL3* gene on 1p31.3 chromosome and inhibits lipoprotein lipase (LPL) and endothelial lipase (EL) activity, thus increasing VLDL and modulating HDL metabolism [114]. In the Dallas Heart Study it was reported that *ANGPTL3* LOF variants were characterized by extremely low TG levels, together with a recessive form of combined hypolipidemia, with very low LDL-C and HDL-C [115]. We previously demonstrated impaired CEC in four patients of three kindreds with very low HDL-C and LDL-C, carrying homozygous or compound heterozygous *ANGPTL3* LOF mutation (p.G400VfsX5, p.I19LfsX22/p.N147X), with absence of plasmatic ANGPTL3. Their HDL showed a reduction in CEC through the various pathways (ABCA1-, ABCG1-, SR-BI-mediated cholesterol efflux), despite the absence of clinical atherosclerosis, probably due to the parallel reduction in LDL-C [116]. Similarly, 66 subjects with familial combined hypolipidemia (FHBL2) carrying the heterozygous or homozygous LOF mutation p.S17* in the *ANGPLT3* gene, showed a significant gene–dose dependent reduction in aqueous diffusion, ABCG1- and SR-BI-mediated CEC, with a negative correlation with the carotid IMT [117].

### 4.2. HDL CEC in Chronic Pathologies

#### 4.2.1. Chronic Kidney Disease

Chronic kidney disease (CKD), with hypertension and diabetes mellitus being the most common causes, has been recognized as a risk factor for CVD independent of other conventional risk factors [118]. Despite CVD being the most important and frequent cause of death of CKD patients globally, the underlying mechanisms are not entirely clear. In this respect, CKD is accompanied by a derangement in lipoprotein metabolism that leads to dyslipidemia. Indeed, not only a reduction in HDL-C levels occurs, but also alterations in HDL structure and composition [119]. Moreover, efforts have been made to investigate a possible role of HDL CEC in the higher CV risk in this population. Previously, was reported that HDL isolated from CKD patients display a reduced CEC [120,121,122].More recently, HDL CEC has been determined in 12 pediatric and 17 young adult patients with CKD stages 3–5, 14 young adult hemodialysis patients and 15 adult renal transplant recipients without associated diseases and matched controls. Only in hemodialysis patients serum CEC was markedly reduced to 85 ± 11% of controls [123]. With this result, authors suggest that an impaired CEC may not play a major role in the pathogenesis of atherosclerosis during the earlier stages of kidney disease and in kidney transplant patients, while it may be important in patients on hemodialysis. This would be in line with much evidence of a profound compositional modification of HDL in this population, which may reduce the atheroprotective functions of HDL [124]. These abnormalities can, in turn, contribute to the progression of CV complications and various other comorbidities, such as foam cell formation, atherosclerosis, and/or glomerulosclerosis, in affected patients. It has also been reported oxidative modifications of apoA-I, mainly on its methionine, tyrosine, or tryptophan residues via the myeloperoxidase pathway, which resulted in the reduced ability of apoA-I to promote cholesterol efflux via ABCA1 [125].

Recently, different results were obtained by Gipson and collaborators, who evaluated serum CEC in healthy subjects, patients with at least one CAD risk factor, patients with established CAD, and in patients with CKD stages G3 to G5. Although healthy subjects, patients with at least one CAD risk factor, and patients with established CAD all showed similar CEC, patients with CKD showed significantly higher CEC. On the other hand, compared to healthy subjects, in all groups the capacity of serum to deliver cholesterol to the liver through SR-BI was reduced [126], suggesting disturbances in the release of cholesterol to the liver for final elimination, the last step of RCT, rather than in CEC, possibly contributing to the increased risk of CAD in patients with CKD.

#### 4.2.2. Obesity

Obesity is a medical condition characterized by excess accumulation of body fat, profoundly affecting health: obese subjects, indeed, usually bear a plethora of pathological conditions, including atherogenic dyslipidemia, with low HDL-C, altered HDL subclass distribution, composition and functions, and increased TG-rich lipoproteins [127]. Interestingly, CEC seems to be inversely associated with the body mass index (BMI), as shown by Talbot and colleagues [128]; moreover, hypoadiponectemia, common in obesity, seems to be a strong predictor of low CEC in adults [129]. Endothelial dysfunction represents a typical feature in subjects with increased CV risk. Decreased ABCA1-mediated efflux was observed in a cohort of 54 overweight or obese women compared to healthy controls, with a parallel increased of apoA-I nitration, which is an independent predictor of endothelial dysfunction [130]. The first-line intervention in obese subjects is represented by weight-loss and lifestyle-changing programs: however, a study conducted upon 52 abdominally obese men with lower CEC compared to matching controls, revealed that after 6 weeks of very low-calories diet, CEC was still impaired, despite a significant weight loss [128]. Similarly, Kjellmo and colleagues considered a similar patient cohort after a diet regimen, reporting lower TG and LDL-C levels, even if the apoB/apoA-I ratio and CEC were unchanged. In those patients, bariatric surgery, able to facilitate a substantial weights loss, which has been demonstrated to decrease mortality from CVD [131], further reduced TG and LDL-C levels but also the apoB/apoA-I ratio, Serum Amyloid A (SAA)/PON1 ratio, and increased HDL-C levels, but had no effect on CEC [132]. Aron-Wisnewsky and colleagues showed that 6 months after Roux-en-Y bypass (RYGBP) surgery HDL-C levels were increased in 34 obese women compared to baseline levels, together with a reduction in CETP activity, reflecting an overall increase in large HDL_2_ subfraction. Consistently, both ABCG1-and SR-BI-mediated plasma CEC were increased [133]. Accordingly, RYGBP surgery and sleeve gastrectomy (SG) surgery were able to increase ABCA1-independent CEC in 37 obese patients with type 2 Diabetes Mellitus (T2DM) also 5 years after the procedure, while other clinical interventions as intensive medical therapy were unable to positively affect CEC [134]. A study conducted by Heffron and colleagues aimed to compare the effects of different bariatric surgical procedures in terms of CEC, by considering 31 severely obese women that underwent RYGB and 36 severely obese women that underwent SG. They observed that 6 months after the procedure, patients that underwent SG displayed increased HDL-C and apoA-I levels, together with increased total and non ABCA1-mediated CEC compared to patients with RYGB. Interestingly, 1 year after SG, CEC was similar to that of subjects with normal BMI, while women that underwent RYGB presented an ameliorated CEC with respect to baseline but still impaired compared to healthy subjects [135].

#### 4.2.3. Diabetes

It is well accepted that both structural and compositional alterations in HDL are associated with diabetes, with an abnormal enrichment in TG and decrease in cholesterol, increased content of SAA, fibrinogen and apolipoprotein C- (apoC) II and III, together with a reduction and glycosylation of apoA-I, apoA-II, apoE, apoM and PON1, leading to a significant alteration of HDL antiatherogenic properties, including their ability to promote cholesterol efflux [136]. Many studies focused on HDL CEC alteration in patients with T2DM, corresponding to about 90% of all cases of diabetes with an increasing prevalence worldwide [137]. A severe reduction in CEC was observed both in plasma and in the interstitial fluid of 35 T2DM patients compared to controls; interestingly, the interstitial fluid is the peripheral compartment in which RCT originates [138]. Notably, Shiu and colleagues observed a strong reduction in plasma preβ-HDL in a cohort of 640 patients with T2DM compared to matched non-diabetic subjects, reflecting a reduction in ABCA1-mediated cholesterol efflux [139]. Consistently, isolated HDL from T2DM subjects displayed a reduced ABCA1 CEC compared to control subjects, probably due to lower HDL levels of serpin family A member 1 (SERPINA-1) that allows apolipoproteins to bind phospholipids [140]. Again, Blanco-Rojo and colleagues conducted a prospective study on 462 participants at the CORDIOPREV study without T2DM at baseline and evaluating the relationship between CEC and T2DM incidence within 4.5 years of follow-up: among them, 106 subjects developed T2DM. Interestingly, an inverse association between HDL cholesterol efflux and T2DM was found, even after normalization to apoA-I and adjustment for parameters related to glucose metabolism [141]. A typical feature of T2DM is the presence of Advanced glycation end (AGE) products, including AGE albumin: Machado-Lima and colleagues demonstrated that glycated albumin isolated from 14 patients with poorly controlled T2DM negatively impacts on cholesterol efflux to apoA-I, HDL_2_ and HDL_3_, mainly by downregulating ABCG1 expression and leading to a greater intracellular cholesterol accumulation [142]. Moreover, a recent study showed an inverse relationship between apoA-I autoantibodies, Ac-terAA1 and AAA1, and CEC, in a cohort of 75 T2DM patients compared to matched healthy controls, probably due to an inverse relationship with apoB-containing lipoproteins [143]. However, other works highlighted a similar [144] or even increased [145] CEC in T2DM patients compared to controls, suggesting a link to the degree of hypertriglyceridemia, usually associated with T2DM, that may act as a potent modulator of CEC by increasing the levels of preβ-HDL, specific cholesterol acceptors for ABCA1- mediated efflux. Despite the predominance of small HDL in plasma of diabetic subjects, however, no significant correlation was found between small HDL particles and CEC, suggesting that T2DM is usually accompanied not only by alteration in HDL subclass distribution but the alteration mainly affect their functionality [146].

Similarly to T2DM, patients with Type 1 Diabetes (T1D) are characterized by an increased CV risk with early onset; interestingly, their HDL-C levels are usually increased compared to healthy subjects, suggesting that, despite the beneficial anti-atherogenic properties of HDL, they cannot be considered a major protective factor in T1D [147,148]. Gourgari and colleagues compared lipoprotein particles and CEC in 78 T1D young patients and healthy controls, highlighting an atherosclerotic-prone phenotype in T1D subjects, with increased LDL-C, smaller HDL size and lower CEC, suggesting that CEC may be considered in CVD risk stratification for T1D [149]. Conversely, Ahmed and colleagues compared HDL size, concentration and CEC in 100 T1D patients compared to 100 controls in a cross-sectional study, showing a reduction in total HDL particles concentration, with a shift towards large HDL particles in T1D subjects and an increased total CEC [150].

#### 4.2.4. Metabolic Syndrome

Metabolic syndrome (MetS) represents a cluster of pathological conditions occurring together, including obesity, hypertension, non-alcoholic fatty liver disease (NAFLD), high plasma glucose, hypertriglyceridemia and dyslipidemia, leading to a two-fold increased risk of CVD and T2DM. Interestingly, subjects with MetS carry not only abnormalities in HDL-C levels but, interestingly, also in their structure and functions [151,152]. It is well established, indeed, that patients with MetS usually present increased levels of small dense HDL and preβ-HDL, together with a reduction in mature HDL particles, as a consequence of altered intravascular HDL remodeling [153]. Consistently, Lucero and colleagues showed an increased ABCA1-mediated CEC in patients with MetS, as a consequence of higher preβ-HDL levels and a slight reduction in LCAT mass, probably reflecting an impairment in RCT [154]. However, a study involving a large cohort of patients presenting at least three criteria of MetS, clearly demonstrated that the increased ABCA1-dependent CEC is unable to counterbalance the reduction in ABCG1- and SR-BI-mediated CEC; accordingly, multivariate analyses suggested a significant association between total CEC and established MetS, independently of age, LDL-C, lipid lowering therapy, smoking or alcohol consumption [155]. Interestingly, Annema and colleagues evaluated CEC of apoB-depleted sera from 552 participants of the CODAM cohort, of subjects with impaired glucose metabolism or T2DM, 97 of which had clinical features of MetS. They showed a similar CEC among subjects with different glucose tolerance categories, highlighting however a reduction in subjects with MetS, independently of the glucose tolerance status [156]. As previously stated, NAFLD is frequently associated with obesity and MetS: CEC was evaluated in 639 participants in the PREVEND study, 226 of which had MetS, considering a Fatty Liver Index (FLI) ≥ 60 as a proxy of NAFLD. Interestingly, multivariable linear regression analyses suggested an inverse association between elevated FLI and CEC [157]. Consistently, Di Costanzo and colleagues confirmed that metabolically-driven NAFLD, unlike genetically-driven NAFLD, is associated with impaired HDL CEC, suggesting an impairment in the anti-atherogenic properties of HDL in subjects with MetS [158]. As stated before, obesity is one of the main common features of MetS: interestingly, subjects with MetS and control subjects underwent a 12 weeks program of weight loss (WL; *n* = 17), weight loss and exercise (WLEX; *n* = 19) or no treatment (*n* = 19), showing that WLEX was more effective compared to WL alone in normalizing the HDL lipidome, particle size, as well as in enhancing serum CEC [159].

#### 4.2.5. Endocrine Disorders

##### Hypogonadism

Low testosterone levels are associated with an increased incidence of CV events [160] but the underlying biochemical mechanisms are not fully understood. The clinical condition of hypogonadism represents a model to unravel the possible role of lipoprotein-associated abnormalities in CV risk, such as the functional capacities of HDL. We recently demonstrated that in cases of genetic and idiopathic hypogonadisms, very low levels of testosterone were associated with an impaired total HDL CEC. This finding was confirmed when the main cellular cholesterol efflux pathways, ABCA1 and ABCG1, were evaluated individually [161]: hypogonadal patients showed lower HDL CEC by both pathways, despite HDL-C levels were in the normal range. The decrement in HDL function may thus suggest that a reduction in the amounts and/or efficiency of either small or large HDL [32,162], or HDL remodeling [163] may occur in hypogonadism. In middle-aged subjects, medically castrated by the gonadotropin-releasing hormone antagonist acyline, HDL-C rises were reported, but not effect was observed on HDL CEC [164]. The same pharmacological castration procedure in young healthy men increased both HDL-C and HDL CEC [165]. Compared to those studies, our findings obtained in naΪf hypogonadal patients indicate a reduction in HDL CEC in a clearly different experimental setting with a consequently different impact on the lipoprotein functional profile. With respect to testosterone supplementation and HDL function, Rubinow and colleagues did not find any differences in HDL CEC after testosterone replacement in older hypogonadal men but testosterone was associated to an altered HDL proteome, i.e., it raised paroxonase-1 and fibrinogen-α-chain and reduced apolipoprotein A-IV, apolipoprotein C-I and paraoxonase 3 [166].

##### Hypothyroidism

Overt hypothyroidism is a well-established risk factor for atherosclerotic CVD [167], partly explained by a profound effect of the disease on lipid and lipoprotein metabolism. In this regard, only a recent study investigated the impact of severe short-term hypothyroidism on HDL particle characteristics, including HDL CEC in 17 patients who have undergone a total thyroidectomy for differentiated thyroid carcinoma [168]. Authors found that during hypothyroidism, despite an increase in HDL and apoA-I levels, HDL CEC was decreased and concluded that the sole increase in HDL cholesterol concentrations does not sufficiently counteract the deleterious effects of the elevation in LDL cholesterol and of the reduced HDL CEC.

##### Polycystic Ovary Syndrome

Women with polycystic ovary syndrome (PCOS) have a high prevalence of CVD risk factors including dyslipidemia. In this regard, few studies investigated the HDL function, measured as HDL CEC, in women with PCOS. Among these, Roe A. and colleagues, in a case-control study conducted in 124 women with PCOS and 67 geographically matched controls, evaluated lipoprotein profile including lipid particle size and number and CEC to better define CVD risk in this population [169]. Authors found an impaired HDL function as compared to controls despite similar HDL-C levels. This result was accompanied by an increase in small LDL particles, both contributing, according to the authors, to the increased CVD risk in women with PCOS. More recently, our research group studied whether ovarian dysfunction in PCOS patients is associated with altered macrophage inflammatory responses and HDL CEC, distinguishing all the main efflux mechanisms, as compared to cycling women in the follicular and luteal phases [170]. Overall, the activation profile of blood-derived macrophages was modulated by the menstrual cycle and macrophage responses to activating stimuli in PCOS patients were blunted compared with cycling women. Interestingly, menstrual cycle did not influence HDL function measured as CEC in healthy women; conversely, total HDL CEC and CEC through the aqueous diffusion process, were significantly impaired in PCOS patients, at least in part, independently of lower HDL-cholesterol levels. This suggests that, in PCOS, dysfunction likely affects particularly large mature HDL. Indeed, HDL qualitative particle modifications are known to be even more important than plasma levels for HDL function, and other authors have reported alterations in HDL size and composition in PCOS. In addition, depletion of estrogen fatty acyl derivatives might be involved in HDL dysfunction observed in PCOS patients [171,172]. We also reported that HDL CEC was positively correlated with both LH and FSH serum levels in PCOS patients, unraveling a relationship between pituitary hormones and HDL function. Dokras and colleagues studied the effects of oral contraceptive pills, the first-line treatment for PCOS, on HDL CEC in 87 obese women with PCOS [173] finding that treatment resulted in multiple effects on lipids including a beneficial increase in HDL CEC. However, the long-term CV impact of these lipid changes in this high-risk population remains unclear.

### 4.3. HDL CEC and Inflammation

#### 4.3.1. Inflammation and Cell Cholesterol Homeostasis

Inflammation is a term covering a wide and complex ensemble of processes due to the activation of cells of the natural and/or adaptive immune system in response to a harmful agent, or something perceived as such, and to tissue damage. The activation of immune cells is associated with the secretion of several soluble mediators and expression of receptors involved not only in the cross-talk with other immune cells but also in the regulation of non-immune cells, like endothelial, epithelial or smooth muscle cells, fibroblasts, adipocytes and hepatocytes. Thus, inflammation is entangled with many other processes related for example to blood pressure, hormonal balance, central and peripheral nervous system function, metabolism. With respect to lipid metabolism, inflammation impacts on the levels and composition of circulating lipoproteins and on cell cholesterol homeostasis through the regulation of cell cholesterol transporters [174,175]. However, such a relationship appears to be bidirectional, as dysfunctional serum lipoproteins, particularly HDL, and cell cholesterol unbalance may be important factors generating or amplifying pro-inflammatory signals [81,176]. Moreover, impaired cholesterol efflux and cholesterol accumulation in dendritic cells promote T cell activation and the loss of immune tolerance [177]. Indeed, expanding clinical data confirm the association between HDL CEC impairment and inflammation in several conditions, such as acute phase reaction and rheumatologic diseases.

#### 4.3.2. HDL CEC Impairment in Inflammatory Condition

##### Acute Phase Reaction

Acute phase reaction (APR) is a systemic host defense response against infectious agents or other noxious insults and often is associated with sepsis. During APR, and particularly in the presence of sepsis, low HDL-C levels are reported, possibly due to increased clearance and reduced synthesis of HDL particles [178]. In addition, profound modifications in HDL composition occur, with increase in SAA and displacement of apoA-I [178], modifications in the relative content in up to one third of HDL-associated proteins and the appearance of proteins normally undetectable [179,180].

Furthermore, HDL CEC is impaired in inflammatory conditions, as demonstrated in experimental models and in clinical studies, often independently of serum levels. Reduced SR-BI- and ABCA1-mediated CEC was reported in inflammatory HDL of healthy volunteers treated with lipopolysaccharide [181]. Moving to real-life situations, we showed that hospitalized patients with APR of various origins presented significantly reduced CEC in models measuring macrophage aqueous diffusion and total efflux, ABCG1- and SR-BI-mediated efflux. Except for the latter pathway, CEC reduction was independent of HDL serum levels [182]. In both the aforementioned studies, CEC impairment was significantly correlated with inflammation markers and SAA-HDL content. Interestingly, increased SAA content was found in HDL as early as 24 h after cardiac surgery [183]. Another small study reported impaired total cholesterol efflux using HDL from septic elderly patients compared to healthy controls [184].

HDL remodeling and CEC impairment were also described in periodontitis, a chronic inflammatory condition of infectious origin [185]. In the same study, an improvement in all parameters was found after periodontitis treatment.

##### Rheumatologic Diseases

Rheumatologic autoimmune diseases are associated with accelerated atherosclerosis and increased CV risk, due to various mechanisms, involving traditional and specific risk factors [186]. Lipid metabolism is in between the two types, as its abnormalities in autoimmune conditions are complex and may differ among diseases and among patients and may vary with disease activity and treatment. In some cases, patients present increased pro-atherogenic lipoproteins and reduced HDL, similarly to the general population at high CV risk. More often, especially during flares of disease activity, the typical lipid profile is that of reduced total cholesterol and LDL, reduced HDL and reduced or normal TG. However, this pattern is not protective in terms of CV risk. For rheumatoid arthritis (RA), the concept of “lipid paradox” was formulated to indicate that, opposite to the general population, patients with low or very low LDL levels have an increased CV risk, the curve between the two variables being U-shaped [187]. More recently, it has been proposed to drop the term “paradox”, based on the increased knowledge on the activity of inflammation both on lipid metabolism and on other pro-atherogenic processes. As previously said, inflammation is associated with profound modifications of lipoprotein levels and quality that, for HDL, translate into impaired anti-inflammatory functions [188] and CEC [189].

HDL CEC impairment has been reported in various rheumatologic autoimmune diseases, such as RA and systemic lupus erythematosus (SLE) [190,191,192,193,194]. In SLE, HDL CEC was inversely related to subclinical carotid atherosclerosis [195]. In our study [191], in RA ABCG1-mediated CEC was specifically impaired and inversely related to the disease activity score DAS28. The mechanisms behind the impairment of HDL protective and anti-atherogenic properties in RA are not entirely known. Data point to changes in HDL composition, with a reduction in antioxidant proteins, such as paroxonase [190] and the increase in proinflammatory molecules, such as SAA [196] or oxidized fatty acids [197]. Few studies have reported reduced CEC in patients with psoriatic arthritis [194,198] and anchylosing spondylitis [199] compared to controls.

Several effects of anti-rheumatic treatment on lipid metabolism have been reported in RA and SLE [200,201,202]. Due to the number of disease-modifying anti-rheumatic drugs (DMARDs) and biologic drugs now available and the various protocols applied, from monotherapy to combination treatments, many different patterns of lipid changes have been reported. In general, anti-inflammatory treatment is associated with reversal of dyslipidemia typical of active autoimmune diseases, thus resulting in an increase in serum TC, LDL-C and HDL-C, and a decrease in Lp(a) levels. This effect seems not to be detrimental, as growing evidence points to CV protective effects of DMARDs and biologic drugs used in the therapy of autoimmune diseases [203]. It is noticeable that if the available data on serum lipid levels changes upon RA treatment are abundant, much less is known about the possible modifications of functional characteristics of circulating lipoproteins. Few are the studies reporting on the modifications of serum lipoprotein quality and function upon the treatment with DMARDs and biologic agents. During the therapy with methotrexate (MTX) and tocilizumab (TCZ), a modification of HDL particle distribution, with an increase in small dense HDL-C particles, a decrease in the medium dense HDL-C particles, and an improvement of anti-inflammatory properties of HDL were reported [204]. Beneficial modifications in HDL particle distribution, but in the absence of functional data, have been described in RA patients treated with rituximab [205]. In patients with early RA, the treatment with various DMARD combinations improved HDL composition (paraoxonase 1 activity, haptoglobin, apoA1, myeloperoxidase) and anti-oxidant capacity, correlated with disease activity reduction [206]. Similar positive modifications in HDL quality were reported for the use of adalimumab and abatacept [207].

Anti-rheumatic treatment is associated with improvement of CEC in rheumatoid arthritis [208,209], in PsA [210] and in juvenile arthritis [211]. We have reported that MTX treatment alone is associated with an increase in ABCG1- and SR-BI-mediated HDL CEC [212]. In the same study, adalimumab treatment was associated with increase in serum HDL levels together with transient modifications of SRBI-mediated CEC (increased) and ABCA1-mediated CEC (decreased), suggesting a maturation shift of HDL particles. The improvement of CEC mediated by mature HDL particles both for MTX and adalimumab is consistent with the report of a block of HDL maturation in inflammatory conditions [182]. TCZ use was associated to improvement in cell cholesterol-related lipoprotein functions, as suggested by a pilot study of ours on RA patients [213], showing that after treatment ABCG1- and SR-BI-mediated CEC are improved, despite HDL serum level stability. Conversely, in a study comparing the effects of MTX alone or in combination with infliximab, HDL CEC, measured by an assay not distinguishing between cell cholesterol efflux pathways, was unchanged [214].

### 4.4. HDL CEC, Aging and Mortality

Atherogenesis is a process beginning in the firsts decades of life and progressing with the years. Thus, several authors analyzed how HDL CEC, and its relationship with atherosclerosis and CVD, may vary during aging.

In this regard, HDL CEC examined in 2282 healthy, young adults aged 24–39 years correlated with vascular markers of subclinical atherosclerosis. Surprisingly, the investigators found an inverse correlation between CEC and FMD and with the Young’s elastic modulus evaluated at the baseline. These data suggest that higher, instead of lower, CEC in this cohort may predict early atherosclerotic alterations. However, after evaluating patients after 7 years of follow-up, this association reversed and resembled that normally observed in adult subjects, where high CEC is a protective marker for CVD risk [215]. The reason of this paradoxical relationship still remains to be elucidated.

When CEC was instead evaluated in aged adults, authors generally demonstrated decreased CEC associated to aging [216,217]. However, our research group, by examining individuals ≥ 80 years and perfectly healthy, found no association with either atherosclerotic burden or with features of plaque vulnerability [218]. In these subjects, CEC was surprisingly higher compared to a cohort of sex-matched younger individuals, leading us to hypothesize that the increased CEC is a favorable process protecting old individuals from CV events and justifying the lack of correlations with atherosclerosis features that we observed.

Concerning the relationship between CEC and mortality, in the prospective observational study including 468 participants of the LUdwigshafen RIsk and Cardiovascular Health (LURIC) study, HDL CEC displayed an inverse relationship with CV mortality, with a reduction of about 27% in the higher CEC quartile both in patients with and without verified CAD, and after adjustment for traditional risk factors [219].

Moreover, in patients with acute ST-segment elevation MI, a stepwise inverse relationship between increasing quartiles of CEC and all-cause mortality at 6 years was detected [220]. However, the most convincing report of the existing link between CEC and all-cause mortality comes from the meta-analysis that was already mentioned above, in which the relative risk of the association between 1SD increase in CEC and all-cause mortality was 0.77 [11].

## 5. Strategies to Increase RCT in Humans

### 5.1. Lifestyle

A growing body of data suggests that improving lifestyle habits, such as adopting a healthy diet, increasing physical activity, and quitting smoking may have a favorable effect on HDL CEC.

A strong consensus indicates that the Mediterranean diet brings significant CV health benefits, as clearly demonstrated by the results of the randomized controlled PREDIMED trial [221]. In a sub-set of the PREDIMED participants, Hernaez and colleagues demonstrated that Mediterranean diet improves HDL CEC, providing a potential mechanism underlying the favorable impact on this diet on CVD health [222]. Among diet components, extra-virgin olive oil and whole grains were the most strongly and independently associated with increased CEC [222]. These latter results support other data indicating that unsaturated fatty acids improve the HDL function [223,224,225]. Interestingly, a study compared the influence of different dietary fats on CEC in individuals with abdominal obesity. Patients were randomized to a 4-week diet rich in saturated fatty acids (cheese or butter) or unsaturated fats ((olive oil (monounsaturated), or corn oil (polyunsaturated)). Consistent with the PREDIMED results, consumption of monounsaturated fat increased HDL CEC, but no change was observed with polyunsaturated [226]. In addition, the diet rich in saturated fats from butter significantly increased HDL CEC compared with saturated fats from cheese, highlighting the importance of food matrix in modulating HDL function.

Conversely, a recent small randomized cross over trial in individuals at high risk of CVD comparing the effects of the consumption of unsaturated fatty acids, walnut or vegetable oils did not detect any differences in HDL CEC [227]. Similarly, the consumption for 12 weeks of alfa-linolenic-reach oil, or either lean or fatty fish did not translate into any HDL CEC improvements in 88 subjects with impaired glucose metabolism [228].

The Mediterranean diet also includes the daily moderate consumption of alcohol that may itself modulate HDL CEC. In this regard it has previously shown that a moderate alcohol consumption may improve the cholesterol efflux ability of HDL via the transporters SR-BI and ABCA1 [229,230,231,232]. On the other hand, long-term ethanol consumption was able to suppress this HDL property, probably related to a depletion of the HDL-associated sphingomyelin [233]. Consistently, plasma HDL from human alcoholics exhibit impaired cholesterol efflux from foam cell macrophages [234].

Beyond diet intervention, physical activity has a well-documented beneficial effect on CV health and is highly recommended for reducing CV risk in the most recent guidelines [235]. This strong relationship has brought researchers to investigate the potential impact of physical activity on HDL antiatherogenic functions, including CEC and the main results have been reviewed by Ruiz-Ramie J.J. and colleagues [236]. All together the results of the available studies provided controversial results and the reason of such variability may be related to the exercise dose, study duration, subjects’ characteristics and concomitant intervention (e.g., caloric restriction or medications). Lately, a convincing result was again obtained on a subset of about 300 participants of the PREDIMED study. It was found that 1 year of real-life levels of physical activity was associated to improvement of several markers of HDL functionality, including CEC [237].

As regards physical activity, also the impact of physical inactivity on HDL CEC has been investigated. A 21-day period of bedrest in healthy male volunteers led to impaired HDL CEC, but the observed change was exclusively related to the lowering effect on HDL-C plasma levels [238].

Among the other modifiable life styles, cigarette smoking is strongly associated to CVD morbidity [239]. The impact of cigarette smoking cessation on HDL function has been poorly explored. A 12-week smoking cessation program in 32 Japanese adults led to a significant improvement of CEC, despite a lack of changes in the levels of HDL-C and apoA-I, potentially providing an additional mechanism explaining why smoking cessation reduces the risk of CVD.

In conclusion, although the majority of the data suggest that lifestyle interventions may improve HDL CEC, many controversies still exist, and additional clinical trials are certainly warranted to shed light on this complex issue.

### 5.2. Nutraceuticals

In moderate hypercholesterolemia, an available alternative to pharmacological therapy is the use of innovative nutritional compounds, the so-called nutraceuticals, as the recent guidelines recommend [235]. Several nutraceuticals have shown significant effects in improving plasma lipid profile, by reducing total cholesterol and LDL-C and by moderately increasing HDL-C with a potential positive impact on CV risk. Moreover, some studies also reported a favorable impact of nutraceuticals on HDL CEC. It has been documented that dietary flavonoids, e.g., anthocyanidins, flavonols, and flavone subclasses influence RCT and HDL function independently of HDL-C in a variety of human populations (e.g., in those who are hyperlipidemic, hypertensive, or diabetic) [240]. In a recent double-blind, randomized, placebo-controlled trial involving 122 hypercholesterolemic subjects receiving 160 mg of anthocyanins twice daily or a placebo (*n* = 61 of each group) for 24 weeks, anthocyanin consumption significantly increased HDL CEC (20.0% increase) as compared to the placebo group (0.2% increase) [241].

In addition, we have recently found that the daily consumption of an innovative pasta enriched in Poliphenols and FIbers enriched in Spores and B-glucans improved the HDL CEC specifically mediated by the transporter ABCG1 [242]. In addition, in another study of ours, we showed that healthy premenopausal women with vitD deficiency who underwent specific supplementation, had improved serum CEC (+19.5%; *p* = 0.003), with a specific increase in the ABCA1-mediated pathway (+70.8%; *p* < 0.001) [243].

We have also demonstrated a complementary beneficial effect of an innovative combination of nutraceuticals containing red yeast rice (monacolin K 3.3 mg), berberine 531.25 mg and leaf extract of *Morus alba* 200 mg (LopiGLIK^®^). Total HDL CEC was improved in 83% of the patients, by an average of 16%, mainly as a consequence of the increase in ABCA1-mediated CEC (+28.5%) [244].

### 5.3. Drugs

Given the clinical relevance of HDL CEC, there is increasing attention given to developing pharmacological approaches to improve this HDL function rather than HDL-C levels, as a potential strategy to reduce CV risk.

The first approach to improve HDL CEC is represented by the CETP inhibitors. Within this context, the TULIP trial was a phase 2 randomized, double-bind, placebo-controlled study that enrolled 364 patients in treatment with a placebo or 1, 5, 10 mg TA-8995, a new CETP inhibitor, or 10 mg TA-8995 combined with 10 mg rosuvastatin [245]. The trial reported an increase in total, non-ABCA1 and ABCA1-dependent CEC in a dose-dependent manner by 38%, 72% and 28% respectively, in patients treated with 10 mg of TA-8995 compared to the placebo group. In addition, a significant increase in pre-β HDL was also observed, that positively correlated with the total and the ABCA1-mediated CEC increase but not with the increase in HDL-C [245]. Similarly, a phase 2 clinical trial evaluated the effect of 12 weeks of treatment with evacetrapib monotherapy, at daily dose of 30, 100 and 500 mg, or in combination with 20 mg atorvastatin, 10 mg rosuvastatin or 40 mg simvastatin [246]. Compared to the placebo, the treatment with evacetrapib alone dose-dependently increased total and non-ABCA1-CEC by 34% and 47%, respectively, while ABCA1-CEC increased in a non–dose-dependent manner. Furthermore, a significant increase in preβ-HDL also occurred, although the mechanism underlying this effect was not known [246]. In addition, a genotype-dependent effect on CEC has been observed for the CETP inhibitor dalcetrapib.

Concerning statins, trials evaluating their impact on CEC have been conflicting. In the phase 2 trial cited above with evacetrapib, study arms involved also statin monotherapy and statin combined with CETP inhibitor [246]. Rosuvastatin and simvastatin significantly reduced total and ABCA1-CEC, whereas atorvastatin did not show a statistically significant effect on any cholesterol efflux pathways. When 100 mg evacetrapib was combined with statin therapy, CEC significantly increased, but this effect was attenuated compared with evacetrapib monotherapy [246]. In the same way, the addition of 10 mg rosuvastatin to 10 mg TA-8995 did not modify CEC compared to 10 mg TA-8995 monotherapy [245]. In addition, a post-hoc analysis of the JUPITER trial evaluated the association between CEC and incident CVD in patients on statin therapy. Rosuvastatin treatment did not lead to a significant change in CEC after 12 months [72]. Overall, it can be stated that the impact of statin therapy on HDL CEC does not appear to be significant, in line with the modest effect of this class of drugs on HDL-C concentration and on subclasses distribution [247].

To a similar conclusion came the results of the trials with niacin. An investigational study evaluated the effect of 2 g/day niacin monotherapy for 16 weeks followed by a washout period of 2 weeks. Niacin administration did not significantly influence CEC compared to baseline; however, stopping niacin resulted in a 9% increase in CEC [248]. Moreover, niacin monotherapy caused proteome remodeling of HDL toward a particle enriched with SAA [248], leading to dysfunctional particles [182].

LDL-apheresis is considered one of the main therapeutic approaches to treat FH, aiming to lower plasma apoB-containing lipoproteins. However, the procedure may also affect plasma HDL-C and apoA-I [249,250]. Nenseter and colleagues demonstrated that in homozygous FH patients, LDL apheresis significantly lowered plasma HDL-C and apoA-I levels, together with a slight reduction in serum CEC compared to controls [250]. Consistently, we have shown that LDL apheresis induced, for at least two days after the procedure, a reduction in AD, ABCA1- and SR-BI-mediated CEC in patients with FH and familial combined hypercholesterolemia [249]. Accordingly, Orsoni and colleagues demonstrated that the acute effect of LDL apheresis in FH patients is the reduction in HDL_2_, HDL_3_ and preβ-HDL [251].

To date, many apoA-I mimetic peptides have been developed and tested in preclinical studies, showing improvements in atherosclerotic-related parameters, including CEC [252]; however, none of them have been tested in human studies so far. On the other hand, various clinical trials evaluated the effect of intravenous apoA-I infusion formulations. As explained in paragraph 3.1.1, subjects with apoA-I_Milano_ were found to be protected against CVD despite the low HDL-C plasma levels. A formulation containing HDL mimetic with recombinant apoA-I_Milano_ (MDCO-216) was tested in a double-blind randomized controlled trial, including 122 subjects with acute coronary syndrome (ACS): ABCA1-mediated CEC increased by 80.4% and 41.6% compared to baseline 2 h and 4 h after the infusion, respectively. However, no significant correlations were detected between CEC and plaque regression among the placebo group and treated patients after 5 weeks of follow-up [253]. A phase 3, double-blind, multicenter randomized clinical trial was aimed at analyzing the effects of 24 weeks of treatment with 8 mg/kg of the HDL mimetic CER-001 or placebo in patients with familial primary hypoalphalipoproteinemia with proven CVD. Plasma CEC was measured at baseline and 2 h after the infusion on week 8 and 24, showing a significant increase in CER-001-treated patients compared to those receiving placebo both at weeks 8 and 24. However, limited effects in terms of arterial wall inflammation and carotid vessel wall plaques extension were observed [254].

Finally, the administration of CSL112, a plasma-derived apoA-I reconstituted with phosphatidylcholine and sucrose, was shown to be associated with a strong increase in ABCA1-mediated CEC [255,256]. In addition, the AEGIS-I multicenter randomized double blind phase 2b trial, aimed to evaluate safety and efficacy of CSL112 administration in 1258 patients with recent myocardial infarction, showed a good level of tolerance in the absence of safety concerns and confirmed the ability of CSL112 to enhance CEC [257].

In conclusion, while some studies suggest that HDL CEC may be pharmacologically modulated, more research is necessary to determine if specifically targeting this parameter will decrease CVD risk.

## 6. Conclusions

HDL are a major player in the modulation of the complex and multifactorial atherosclerotic process underlying CVD, through their anti-inflammatory and anti-oxidant properties, and through their ability to promote cell cholesterol efflux. Much attention is given at present to the evaluation of HDL function, which does not always mirror HDL plasma levels. A growing body of data is currently available on HDL CEC, the ability of HDL to promote cell cholesterol efflux through various efflux pathways, in healthy subjects and in several pathological conditions. Moreover, the molecular mechanism underlying CEC modifications are being explored, as well as the role of this parameter as a CV risk marker and therapeutic target. There is substantial evidence that CEC is a valuable tool to predict CV risk in many patient populations and that its improvement following lifestyle amelioration or after treatment with various nutraceuticals or drugs might be beneficial. However, further studies are needed to clarify still existing controversies and to understand the discrepancies, due for example to the various methods applied for CEC measurement.

In conclusion, these aspects together suggest that HDL are still a promising area of investigation but strategies for identifying efficacy should move to HDL function measurement, e.g., CEC, instead of levels.

## Figures and Tables

**Table 1:** Results of the main cross-sectional studies examining the association between high-density lipoprotein (HDL) cholesterol efflux capacity (CEC) and cardiovascular (CV) risk.

Cross-Sectional Studies			
Study	Study Population	Main Findings	OD/HR/r and *p* Value
Khera A.V. et al., 2011 [37]	203 healthy volunteers; 442 patients with and 351 without angiographically confirmed CAD	HDL CEC was a strong inverse predictor of CAD also after adjustment for HDL-C ^1^or apoA-I levels ^2^	^1^ OR: 0.75; *p* = 0.002 ^2^ OR: 0.75; *p* = 0.002
Ishikawa T. et al., 2015 [60]	182 patients with and 72 without CAD;	HDL CEC, but not HDL-C or apoA-I levels, was a significant predictor of CAD.	OR: 0.23; *p* = 0.037
Ogura M. et al., 2016 [61]	227 HeFH patients of which 76 had ASCVD	Increased HDL CEC was associated with decreased risk of ASCVD even after the addition of HDL-C level as a covariate and after adjustment for age, sex and traditional CV risk factors	OR: 0.95; *p* < 0.05
Thakkar H. et al., 2020 [64]	150 ACS patients; 110 controls	HDL CEC was associated with a higher OR of ACS even after adjustment for confounding factors.	OR: 0.49; *p* = 0.006
Favari E. et al., 2013 [65]	167 healthy subjects	ABCA1-dependent CEC was inversely correlated with PWV	r = −0.183; *p* = 0.018
Vigna G. B. et al., 2014 [66]	20 subjects with HAL; 20 controls	ABCG1-CEC was directly correlated with the FMD	r = 0.377; *p* < 0.05
Josefs T. et al., 2020 [71]	574 subjects from CODAM cohort with T2DM and CVD	HDL CEC was not associated with either markers of atherosclerosis cIMT and EnD, nor with CVD or CVE	*p* = 0.332
Li X.M. et al., 2013 [70]	Cohort A: Stable Angiographic Case—Control Cohort (*n* = 1150); Cohort B: Outpatient Case—Control Cohort (*n* = 577)	Higher CEC was paradoxically associated with increased risk of myocardial infarction/stroke^1^ and major adverse CVE^2^	^1^ HR: 2.19 ^2^ HR: 1.85

ASCVD: atherosclerotic cardiovascular disease; ACS: acute coronary syndrome; CAD: coronary artery disease; cIMT: carotid intima-media thickness; CEC: cholesterol efflux capacity; CV: cardiovascular; CVD: CV disease; CVE: CV event; EnD: endothelial dysfunction; FMD: flow mediated dilation; HAL: hyperalphalipoproteinemia HDL: high density lipoprotein; PWV: pulse wave velocity; T2DM: type 2 diabetes mellitus.

**Table 2:** Results of the main longitudinal studies examining the association between HDL CEC and CV risk.

Longitudinal Studies			
Study	Study Population	Main Findings	OD/HR/r and *p* Value
Rohatgi A. et al., 2014 [58]	2924 subjects free from CVD	HDL CEC was inversely associated with the incidence of CV events after adjustment for traditional risk factors.	OR: 0.33
Saleheen D. et al., 2015 [59]	1745 initially healthy subjects who later developed fatal or non-fatal CHD; 1749 controls	HDL CEC was inversely associated with incidence of CHD events after the adjustment for CV risk factors and HDL-C.	OR: 0.64
Patel P.J. et al., 2013 [62]	23 subjects with CAD and EF < 50%; 46 control subjects without CAD and EF > 55%	Low HDL CEC was a significant risk factors for HF	OR: 2.1 *p* = 0.03
Khera A.V. et al., 2017 [72]	314 subjects with CVD; 314 controls	On-statin HDL CEC was inversely associated with the incidence of CVD ^1^, although HDL particle number emerged as the strongest predictor ^2^.	^1^ OR: 0.62 *p* = 0.02 ^2^ OR:0.51 *p* < 0.001
Ritsch A. et al., 2015 [73]	2450 healthy subjects undergoing coronary angiography	Inverse correlation between CEC and CV mortality in a fully adjusted model that included traditional CV risk factor.	HR for Q4 vs. Q1: 0.64
Chindhy et al., 2018 [74]	2895 subjects without baseline CVD; 210 of them with baseline CKD	No significant interaction between CEC and CKD on associations with ASCVD ^1^ and total CVD ^2^.	^1^ HR: 1.30 *p* = 0.01 ^2^ HR: 1.15 *p* = 0.05
Javaheri et al., 2016 [75]	35 patients with CAV 1 year after heart transplantation	Reduced CEC was independently associated with disease progression and mortality in CAV patients.	OR: 0.35 *p* = 0.048
Kopecki et al., 2017 [76]	1147 patients with T2DM and undergoing hemodialysis	No association between CEC and CVD mortality ^1^, cardiac events ^2^, and all-cause mortality ^3^.	^1^ HR: 0.96 *p* = 0.42 ^2^ HR: 0.92 *p* = 0.11 ^3^ HR: 0.96 *p* = 0.39
Liu et al., 2016 [77]	1737 patients with CAD	CEC was an independent factor to predict all-cause ^1^ and CV mortality ^2^ in patients with CAD	Q4 vs. Q1 ^1^ HR: 0.24 *p* = 0.001 ^2^ HR: 0.17 *p* = 0.001
Mody et al., 2016 [78]	1972 patients with/without CAD	CEC was inversely associated with ASCVD among subjects with CAC ^1^, FH ^2^ and elevated hsCRP ^3^.	^1^ HR: 0.40 ^2^ HR: 0.31 ^3^ HR: 0.37
Soria-Florido et al., 2020 [79]	167 patients with ACS; 334 controls	CEC was inversely associated with ACS incidence ^1^ and MI ^2^.	^1^ OR: 0.58 ^2^ OR: 0.33
Ebtehaj S. et al., 2019 [71]	351 subjects with CVD developed during follow-up; 354 controls	CEC was significantly associated with the future development of CVD events independently of HDL-C and ApoA-I plasma levels	OR: 0.73 *p* < 0.001
Riggs K.A. et al., 2019 [80]	2643 health subjects with < 65 years	GlycA was directly associated with HDL-C and ApoA-I, while it was inversely correlated with CEC	HR for Q4 vs. Q1: 3.00
Annema et al., 2016 [82]	495 patients that underwent renal transplantation	CEC was not associated with future CV mortality ^1^ or all-cause mortality ^2^, while it was found to predict graft failure ^3^.	^1^ HR: 1.014 *p* = 0.92 ^2^ HR: 0.908 *p* = 0.35 ^3^ HR: 0.428 *p* = 0.001
Shea S. et al., 2019 [83]	465 cases with incident CV events; 465 controls	CEC was significantly associated with lower odds of CVD ^1^, higher CEC was associated with lower risk of incident CHD ^2^.	^1^ OR = 0.82 *p* = 0.031 ^2^ OR = 0.72 *p* = 0.007
Garg p.K. et al., 2020 [84]	1458 patients that developed incident clinical or subclinical PAD during 6 years of follow-up	High CEC was not significantly associated with incident clinical PAD ^1^, or subclinical PAD ^2^	^1^ HR: 1.25 ^2^ HR: 1.02

ACS: acute coronary syndrome; ASCVD; atherosclerotic cardiovascular disease; CAC: coronary artery calcium; CAV: cardiac allograft vasculopathy; CEC: cholesterol efflux capacity; CHD: coronary heart disease; CKD: chronic kidney disease; CV: Cardiovascular; CVD: CV disease; HDL-C: high density lipoprotein cholesterol; HF: heart failure; hs-CRP: high-sensitivity C-reactive protein; MI: myocardial infarction; PAD: peripheral artery disease.
